# Impact of Carbohydrate-Electrolyte Beverage Ingestion on Heart Rate Response While Climbing Mountain Fuji at ~3000 m

**DOI:** 10.1155/2017/3919826

**Published:** 2017-07-10

**Authors:** Masahiro Horiuchi, Junko Endo, Koichi Kondo, Tadashi Uno, Mayuko Morikawa, Hiroshi Nose

**Affiliations:** ^1^Division of Human Environmental Science, Mount Fuji Research Institute, Kamiyoshida 5597-1, Fujiyoshida, Yamanashi 4030005, Japan; ^2^Mount Fuji Climbing School, Asahi 4-1-7, Fujiyoshida, Yamanashi 4030012, Japan; ^3^Department of Sports Medical Sciences, Shinshu University Graduate School of Medicine, Asahi 3-1-1, Matsumoto, Nagano 3908621, Japan; ^4^Institute for Biomedical Sciences, Asahi 3-1-1, Matsumoto, Nagano 3908621, Japan

## Abstract

We sought to investigate whether carbohydrate-electrolyte beverage ingestion reduced heart rate (HR) in twenty-three healthy young adults while climbing Mount Fuji at a given exercise intensity. Twenty-three healthy adults were randomly divided into two groups: the tap water (11 males [M] and 1 female [F]) and the carbohydrate-electrolyte group (10 M and 1 F). HR and activity energy expenditure (AEE) were recorded every min. The HRs for the first 30 minutes of climbing were not significantly different between the groups [121 ± 2 beats per min (bpm) in the tap water and 116 ± 3 bpm in the carbohydrate-electrolyte]; however, HR significantly increased with climbing in the tap water group (129 ± 2 bpm) but showed no significant increase in the carbohydrate-electrolyte group (121 ± 3 bpm). In addition, body weight changes throughout two days ascending and descending on Mount Fuji were inversely related to changes in resting HR. Further, individual variation of body weight changes was suppressed by carbohydrate-electrolyte drink. Collectively, carbohydrate-electrolyte beverage intake may attenuate an increase in HR at a given AEE while mountaineering at ~3000 m compared with tap water intake.

## 1. Introduction

Mount (Mt.) Fuji (~3776 m) is the highest mountain in Japan. More than 250,000 tourists climb to its peak every summer [1], but the number has been increasing since the mountain was accredited as a World Heritage Site in 2013. More than 400 tourists every year visit the clinic located at 3,000 m for their health issues, and 65% of them show symptoms of acute mountain sickness (AMS) [1]. Since there has been a possibility that AMS is frequently accompanied by dehydration [2], and intravenous saline infusion improves dehydration status [3], the maintenance of euhydration using appropriate beverage intake while climbing may help to prevent AMS. In addition, individual variance of exercise performance under hypoxia with hypohydration was greater compared to euhydration, suggesting that prevention of individual variance may also be important during exercise at high altitude.

Carbohydrate-electrolyte (CE) solution intake has been reported to improve thermoregulatory responses, thereby attenuating an increase in heart rate (HR) at a given exercise intensity through accelerated rehydration during sports events at the sea level compared with tap water (TW) [4–7]. However, there have been few studies that examine this issue during mountaineering because there was no device to measure exercise intensity at high altitude.

Recently, calorimeters equipped with triaxial accelerometers and barometers (to measure kinetic and potential energies, resp.) have become commercially available [8, 9]. Using this equipment, we were able to evaluate the effects of beverage intake on HR at a given intensity during mountaineering [8].

In the current study, we measured HR and activity energy expenditure (AEE) in young adults while climbing Mt. Fuji to a 3000 m altitude, during which they drank CE or TW freely depending on their assigned test group. Then, we compared HR responses at a given intensity between the groups. We hypothesized that HR at a given intensity would be reduced in the CE group compared with the TW group during climbing mountain at high altitude. It was also hypothesized that CE ingestion can prevent individual variance of body weight loss as an indirect indicator of dehydration status.

## 2. Methods

### 2.1. Participants

Twenty-three healthy adult participants were randomly divided into two groups: the TW group (*n* = 12, 11 males [M] and 1 female [F]), which consumed TW (0 kcal energy, 0 g carbohydrate, protein, fat, sodium, potassium, calcium, and magnesium), and the CE group (*n* = 11, 10 M and 1 F), which consumed a CE solution (25 kcal energy, 6.2 g carbohydrate, 0 g protein, 0 g fat, 49 mg sodium, 20 mg potassium, 2 mg calcium, and 0.6 mg of magnesium per 100 ml). Notably, we did not explain the differences in nutrient composition between TW and CE solution, meaning that the subjects could know only differential taste. All subjects were free from any cardiovascular diseases and have not been engaging in regular physical activities. Additionally, this was the first attempt for all subjects to climb Mt. Fuji, and none of the subjects were exposed to an altitude higher than 1500 m within 6 months prior to the study. All the procedures were approved by the institutional ethical committee according to the guidelines of the Declaration of Helsinki. The physical characteristics of the participants are shown in Table 1.

### 2.2. Procedure

The participants arrived at the parking area by car at ~13:00 on July 6, 2014 to climb Mt. Fuji (2305 m) after consuming light lunch ~1 h before their arrival. After they emptied their bladders, we measured their body weight with their clothes and boots on using a 50 g resolution scale (UC-321, A&D Instruments, Tokyo, Japan). We also separately measured the body weight of the participants in the nude, which excluded the weight of their clothes, boots, backpacks (weighing ~7 kg), jackets, sweaters, snacks, and water bottles.

Then, to estimate maximal aerobic capacity, the participants underwent a graded walking test without their backpacks. Briefly, after a baseline measurement at rest in the standing position for 3 min, participants walked for 3 min on flat and paved areas at 3 graded subjective velocities (slow, moderate, and fast), during which AEE was measured with a calorie meter equipped with a triaxial accelerometer and barometer (JD Mate, Kissei Comtec, Matsumoto, Japan) fastened on the left side of their waists. Heart rate (HR) was measured with a commercial wearable HR monitor (RS 800CX, Polar Electro Japan, Tokyo, Japan) with every 5 sec and calculated into every 1-min data.

Oxygen consumption ($\dot{V}O_{2}$) rates during the tests were determined from the values of a triaxial accelerometer and barometer according to the equation reported previously [8, 9]. We determined the peak aerobic capacity for walking (${W\dot{V}O}_{2peak}$) from the last consecutive three values of fast walking and the adopted peak HR (HR_peak_) at ${W\dot{V}O}_{2peak}$. Since HR_peak_ was lower than the age-predicted maximal HR, that is, 220-age, we estimated $\dot{V}O_{2peak}$ (${E\dot{V}O}_{2peak}$) from the relationship between HR and $\dot{V}O_{2}$ for 9 min by extrapolating HR to the age-predicted maximal HR during the test. ${W\dot{V}O}_{2peak}$ and ${E\dot{V}O}_{2peak}$ were presented as ml min^−1^ per body weight in the nude (kg), assuming that the weight of the clothes worn by the participants during the tests was ~2 kg.

**Figure 1:**
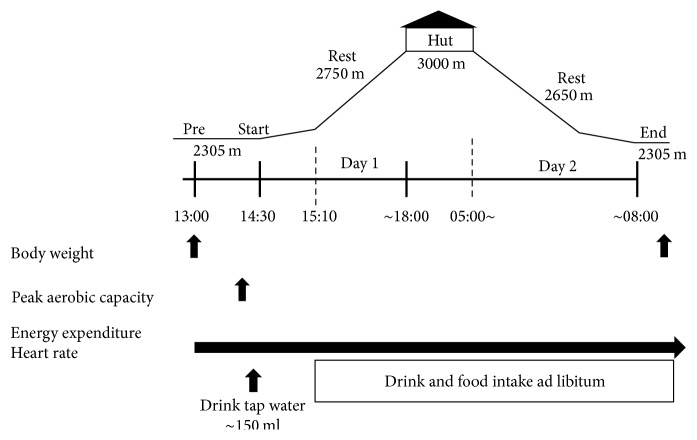
Profile of the present study. Participants consumed tap water or a carbohydrate-electrolyte beverage freely throughout the two days of the study except for the first 40 min before beginning to climb up the mountain on the first day, during which time we measured their body weight and peak aerobic capacity. In addition, we measured their energy expenditure and heart rate continuously while climbing up and down the mountain. Then, after climbing down the mountain, we measured body weight again. The measurements are shown in the procedure of the text.

As shown in Figure 1, after the measurements of 9 min of graded walking test, the participants were allowed to drink a tap water ~150 ml, began to climb the mountain at ~14:30, reached a lodge (~3000 m) at ~18:00, rested at the altitude about ~2750 m, stayed overnight at the hut, started to climb down the mountain at ~05:00 the next morning with a short period rest (2650 m), and returned to the parking area (2305 m) at ~08:00. Throughout two days, trekking, the party of subjects ascended and descended together. During the period, excluding the stay at the lodge, HR and AEE were recorded every min. To determine AEE, we calculated ${\dot{V}O}_{2}$ by multiplying a sum of kinetic and potential energies (measured by accelerometry and barometry, resp.) with a sum of body, clothing, shoes, and baggage weight according to the equation reported previously [8]. Then, the ${\dot{V}O}_{2}$ value was converted to kJ based on a previous study [8, 9]. Immediately after returning to the parking area, we asked participants to empty their bladders and measured their body weights by 50 g scales, in the nude as we did before their climbing (UC-321, A&D, Tokyo, Japan). Throughout the mountaineering, participants were allowed to eat freely including lunch, snacks, drinks (either TW or CE depending on their assigned group), and dinner at the lodge prepared by us; their consumption amounts were recorded. As it has been reported that it took ~30 min until an observation of homogeneously distributed body fluid after drinking water [10], the subjects were asked not to take any drink or foods during this period. Thus, we confirmed no drink or foods during the first 40 min of climbing as well as no evacuation throughout two days of trekking. Their total energy intake and the nutritional composition of their consumed food throughout mountaineering were calculated using a software package (Excel Eiyo-kun, Kenpakusha Co., Ltd., Tokyo, Japan) [8]. The periods when AEE was less than 15% of $\dot{V}O_{2peak}$ obtained from the 9-min graded walking test were regarded as resting periods according to a previous study [8].

Arterial O_2_ saturation (SpO_2_) was measured by pulse finger oximeter (MMI SB-100, Muranaka Medical Instruments Co. Ltd., Osaka, Japan). Symptoms of acute mountain sickness (AMS) were also evaluated by the Lake Louise Questionnaire (LLQ) scoring system and the criteria for AMS were defined as follows: LLQ was ≧3 with headache [11]. Values of SpO_2_ and LLQ score were assessed before ascending (2305 m), at rest during climbing (2750 m), at the hut before sleep and after wake-up (3000 m), and after descending, respectively. Then, the nadir (SpO_2_) and the highest score (AMS) were taken as indices of hypoxemia and severity of AMS.

### 2.3. Statistical Analysis

**Figure 2:**
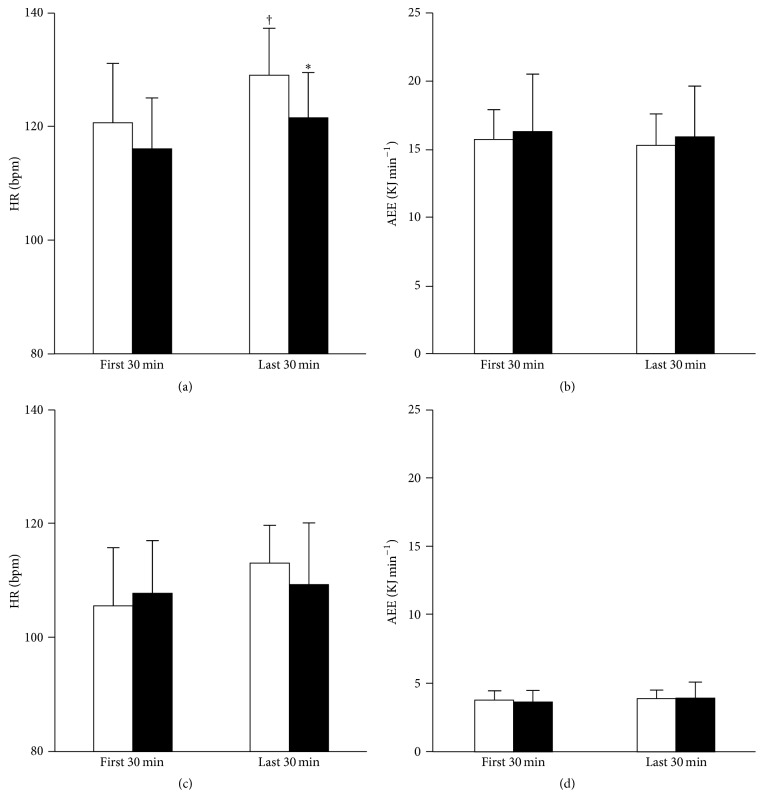
Heart rate (HR) and activity energy expenditure (AEE) for the first and last 30 min of climbing of the total ~210 min during exercise (a and b) and at rest (c and d) on the first day. The means and standard deviation bars for the 12 participants in the TW group and those for the 11 participants in the CE group are shown. White bars indicate the TW, and black bars indicate the CE group, respectively. ^*∗*^*P* < 0.05 between the TW and CE groups within the last 30 min of climbing. ^†^*P* < 0.05 between the first and last 30 min of climbing within the TW group.

The data are expressed as the mean ± standard deviation (SD). A commercial statistical software package was used for all the analyses (Sigma Stat ver. 3.5, Hulinks, Chicago, IL, USA). An unpaired *t*-test was used to examine the significant differences in the physical characteristics, dietary data, and all the physiological values of the participants before climbing the mountain between the groups (Table 1). A two-way (groups × time) analysis of variance for repeated measures was used to examine the significant effects of group (TW and CE) and time (the first and last 30 min of climbing on the first day) on AEE and HR and their interactive effects (Figure 2). A Bonferroni post hoc test was conducted when any *F* values are *P* < 0.05, for further analysis. A Chi-square tests were used to assess numbers of participant's distribution with or without AMS. Pearson correlation coefficient was used to predict a relation between changes in body weight and HR throughout two days of trekking. A *P* value less than 0.05 was used as statistical significance.

## 3. Results

Table 1 shows the physical characteristics, HR at rest, HR_peak_, $W\dot{V}O_{2}$, and $E\dot{V}O_{2peak}$ of the participants during the walking test before climbing the mountain. Nadir values of SpO_2_, individual body weight changes between preascending and postdescending, and a total amount of fluid and energy intake throughout two days were also shown in Table 1. There were no significant differences in the variables between the groups. Similarly, there are no significant differences in detailed nutrients of meals on days 1 and 2 between groups. In regard to nutrients of drinks, although they are natural, amounts of carbohydrate and sodium of CE group were greater on both days, while amounts of protein and fat of both groups are zero (Table 2).

Figures 2(a) and 2(b) show HR and AEE while climbing to the lodge for the first and last 30 min of the first day. Although AEE did not change from the first to the last 30 min of climbing in either group (*P* > 0.05), HR in the TW group for the first 30 min (121 ± 2 beats min^−1^) significantly increased for the last 30 min (129 ± 2 beats min^−1^, *P* = 0.002), whereas HR in the CE group did not reach statistical differences between first 30 min (116 ± 3 beats min^−1^) and last 30 min (121 ± 3 beats min^−1^, *P* > 0.05). In addition, HR in the TW group was significantly higher than those in the CE group for the last 30 min (*P* = 0.031). As shown in Figures 2(c) and 2(d), there was no significant difference in resting periods during ascending, that is, AEE less than 15% of VO_2peak_, HR, or AEE between the first and last 30 min of exercise or between the TW group and the CE group (*P* > 0.05, resp.).

The average HR and AEE while descending on the second day were 105 ± 12 beats min^−1^ and 7.61 ± 1.23 kJ min^−1^ in the TW group and 106 ± 12 beats min^−1^ and 7.23 ± 1.02 kJ min^−1^ in the CE group, respectively, showing no significant difference between the groups (*P* > 0.05, resp.).

**Figure 3:**
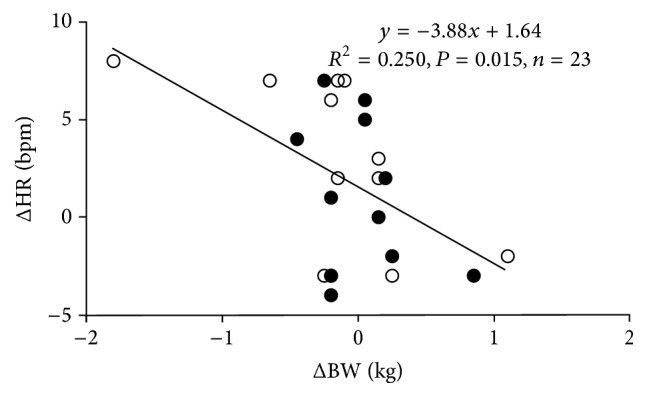
Relationship between changes in body weight and HR when the data are pooled. Changes in these values are calculated by the difference between at rest before ascending and after descending. Open circles indicate the TW and closed circles indicate the CE group.

Figure 3 shows the relationship between the changes in body weight and HR at resting condition from the values before ascending on the first day to the values after descending on the second day. We found that body weight changes significantly correlated with changes in HR at resting condition when the data were pooled from the two groups (*R*^2^ = 0.250, *n* = 23, *P* = 0.015). In addition, although the changes in body weight were −154 ± 193 g in the TW group and 22 ± 106 g in the CE group (see Table 1) with no significant difference in mean values between groups (*P* = 0.441), the standard deviation (SD) was significantly lower in the CE group than in the TW group (*F*[11,12] = 3.581, *P* < 0.01). Despite this significance, the change in HR was 2.6 ± 1.3 beats min^−1^ in the TW group and 1.2 ± 1.2 beats min^−1^ in the CE group, which shows no difference in the SD (*F*[11, 12] = 1.261; *P* > 0.05) or mean values (*P* = 0.432).

The numbers of subjects who reached the criteria of symptoms of AMS were 3 of 11 in the CE groups (27.2%) and 5 of 12 in the TW group (41.7%) with no statistical differences in variance by Chi-square test (*P* > 0.05).

## 4. Discussion

In the present study, we found that (1) CE ingestion, more than TW ingestion, attenuated an increase in HR at a given exercise intensity during the last 30 min of climbing on the first day, (2) the greater body weight loss after two days of climbing caused a greater increase in resting HR, and (3) the interindividual changes in body weight after two days of ascending and descending were within a more narrow range of the variation in the CE group than in the TW group.

The detailed mechanism of the attenuated increase in HR for the last 30 min of climbing on the first day in the CE group remains unknown; however, it has been suggested that CE ingestion accelerates the recovery of dehydration due to a higher retention of the consumed fluid in the extracellular fluid/plasma volumes while sweating, which, otherwise, suppresses cutaneous vasodilation via baroreflexes, reduces heat dissipation, and increases body temperature, which then increases HR by directly stimulating the cardiac pacemaker [12, 13]. Thus, the attenuated increase in HR due to CE ingestion might be caused by the attenuated increase in body temperature with a greater recovery of plasma volume [4]. Additionally, previous animal experiments demonstrated that insulin binds to various segments of nephrons to accelerate the renal Na^+^ reabsorption rate [14, 15]. This may indicate that insulin secretion with carbohydrate intake in meals, for example, the CE solution that is used in the present study, would accelerate Na^+^ and body fluid recovery to attenuate voluntary dehydration. Although speculative, lower HR in the CE groups may be accounted for these effects of carbohydrate and sodium. Because the increase in HR during exercise is known to correlate with the rating of perceived exertion [16], the participants in the CE group likely felt less strain compared with those in the TW group even though they climbed the mountain at the same AEE and speed.

We found no significant difference in HR and AEE between groups while climbing down the mountain on the second day, probably caused by less dehydration due to the shorter period and lower intensity of exercise than on the first day; however, there was a significant correlation between the changes in HR and body weight (Figure 3), suggesting that even a small amount of dehydration increases HR [17, 18]. In addition, the smaller range of interindividual variations in body weight loss in the CE group than in the TW group suggests that CE ingestion recovers plasma volume at a higher probability than TW ingestion. It should be noted that one participant showed a marked body weight reduction and HR increase, resulting in a considerable effect on the significant relationship (Figure 3). However, it may be difficult to detect whether the values of this participant are outlier or not. In addition, a previous laboratory study reported that hypoxic exposure with hypohydration caused a greater individual variance in physiological responses compared to normoxic condition and/or euhydration status [2]. Although future studies should be required with a larger sample size, we analyzed the data using all participants. Since we assessed body weight only before and after descending, other potential factors that may affect body weight changes, such as regulatory systems of body fluid, including renin-angiotensin-aldosterone system, and arginine vasopressin should be considered [3, 19]. However, we tried to compare body weight and HR changes at the same altitude, that is, at the 5th station ~2305 m as we sought to rule out effects of different altitude, particularly, HR changes even at resting condition. Thus, the maintenance of euhydration using fluid ingestion may be useful to prevent an increase in HR during mountaineering, and CE ingestion is more recommendable than TW ingestion to attain the desired purpose.

We did not find any differences in symptoms of AMS and SpO_2_ between groups. As AMS is related to hypoxemia, that is, lower SpO_2_ [20–22], no differences in SpO_2_ between groups may less affect symptoms of AMS between groups though future studies on how CE drinking may affect these symptoms and/or physiological responses should be warranted.

There are several methodological considerations to interpret our results. First, in addition to relative small sample sizes in this study, we recruited different subject's group, so, the effect of different group on physiological responses could not be completely ruled out. However, as it was impossible to control environmental conditions on the field study, we decided to conduct this study for different group on the same day. Furthermore, since there are no differences in physical characteristics and cardiorespiratory variables, our main conclusions may not be strongly affected. Second, each subject consumed fluids and food ad libitum; thus it should be noted to consider other potential effects, for example, energy intake in both fluids and foods and evacuation. However, we confirmed that participants did not evacuate and their total amount of fluids and energy intake showed no differences between groups. Finally, to clarify the underlying mechanisms and potential effect for preventing AMS, though we must acknowledge future study is required, our results may have a potential role for climbers to ingest appropriate drink.

## 5. Conclusions

TW ingestion significantly increased in HR during last 30 min of climbing compared to the first 30 min, while CE ingestion suppressed an increase in HR at a given exercise intensity more effectively. Thus, CE ingestion might be an appropriate strategy to decrease the cardiovascular strain during mountaineering.

## Figures and Tables

**Table 1:** Physical characteristics of subjects and physiological responses between two groups.

	TW	CE	*P* value
	*n* = 12 (11 men, 1 woman)	*n* = 11 (10 men, 1 woman)	
Age, years	28.4 ± 1.9	29.0 ± 1.5	0.810
Height, cm	173.6 ± 1.7	171.6 ± 2.9	0.559
Weight, kg	66.4 ± 2.9	66.4 ± 2.8	0.992
BMI, kg (m^2^)^−1^	22.0 ± 0.8	22.5 ± 0.7	0.616
Resting HR, beats min^−1^	92 ± 2	89 ± 3	0.404
HR_peak_, beats min^−1^	149 ± 4	142 ± 3	0.134
$W\dot{V}O_{2}$, ml kg min^−1^	25.6 ± 1.0	23.8 ± 0.7	0.162
$E\dot{V}O_{2peak}$, ml kg min^−1^	37.0 ± 2.4	36.5 ± 1.9	0.870
SpO_2nadir_, %	86.6 ± 2.6	86.9 ± 2.6	0.767
Body weight changes, g	−154 ± 193	22 ± 106	0.441
Total amount of fluid intake, ml	1280 ± 113	1127 ± 100	0.327
Total amount of energy intake, kJ	5266 ± 420	5506 ± 314	0.656

Values are mean ± standard deviation (SD). TW, tap water group; CE, carbohydrate-electrolyte solution group; BMI, body mass index; HR_peak_ and $W\dot{V}O_{2peak}$, peak heart rate and peak aerobic capacity determined by the graded walking test; $E\dot{V}O_{2peak}$, peak aerobic capacity estimated from the age-predicted maximal heart rate; SpO_2_, arterial O_2_ saturation.

**Table 2:** Detailed nutrients in meals and drink on days 1 and 2 between TW and CE group.

	TW	CE			*P* value
	*n* = 12 (11 men, 1 woman)	*n* = 11 (10 men, 1 woman)			
*Day 1*					
Meals					
Protein, g	26 ± 4	24 ± 4			0.330
Fat, g	25 ± 12	21 ± 9			0.420
Carbohydrate, g	169 ± 45	151 ± 23			0.236
Sodium, mg	1755 ± 276	1766 ± 283			0.928
Drink					
Protein, g	0	0			—
Fat, g	0	0			—
Carbohydrate, g	0	68 ± 21			—
Sodium, mg	0	500 ± 154			—
*Day 2*					
Meals					
Protein, g	4 ± 1	4 ± 1			0.293
Fat, g	3 ± 2	4 ± 2			0.754
Carbohydrate, g	24 ± 11	25 ± 9			0.720
Sodium, mg	248 ± 79	307 ± 105			0.136
Drink					
Protein, g	0	0			—
Fat, g	0	0			—
Carbohydrate, g	0	7 ± 3			—
Sodium, mg	0	52 ± 19			—

Values are mean ± SD. Note that “—” marks indicate without statistical analysis as TW does not contain all nutrients and CE does not contain “protein” and “fat.”
